# Stem cell therapy as a potential treatment option for psoriasis^⋆^

**DOI:** 10.1016/j.abd.2021.10.002

**Published:** 2022-05-27

**Authors:** Piyu Parth Naik

**Affiliations:** Department of Dermatology, Saudi German Hospitals and Clinics, Opposite Burj Al Arab, Dubai, United Arab Emirates

**Keywords:** Interleukins, Psoriasis, Stem cells, Therapy

## Abstract

Psoriasis is a chronic inflammatory dermatological disorder characterized by white scales and clearly demarcated erythematous plaques. The prevalence of psoriasis varies from country to country and can occur at any age, implying that ethnicity, environmental factors, and genetic background all play a role in its onset. According to the World Psoriasis Day Consortium, 125 million people globally and 2%–3% of the overall community have psoriasis. The introduction of biological treatments has revolutionized the treatment of moderate to severe psoriasis. These novel drugs, particularly those targeting interleukin (IL)-17 and IL-23p19, can help most patients with psoriasis achieve clear or virtually clear skin with satisfactory durability. Nevertheless, none of these modern treatments are not entirely remedial in their current form, and alarmingly, a limited but growing proportion of patients with severe psoriasis are not responding satisfactorily to currently available treatments. Stem cell therapy, including regulatory T-cells, hematopoietic stem cell transplantation, and mesenchymal stromal cells, has been used in patients with recalcitrant psoriasis. This review discusses the stem cell treatments available for psoriasis.

## Introduction

Psoriasis is a chronic autoimmune dermatological disease characterized by scaly white or erythematous plaques, with a predilection for the scalp, genitalia, lumbosacral area, and extensor surfaces of the limbs. It affects 2%–3% of the global population, namely about 125 million individuals worldwide. Psoriasis affects people of all ages but is most common in those aged 15–25 years and can lead to psoriasis arthritis. It is more common in Caucasians than in other ethnic groups and is more frequent at greater latitudes. Although psoriasis is classified as an autoimmune disease, there is no evidence to implicate autoantigens; nonetheless, there is evidence that the disease has a genetic predisposition.^1^ Genetic and environmental factors can influence the age of onset of psoriasis. For example, the presence of the Human Leukocyte Antigen (HLA)-C*06 alleles has been linked to a younger age of onset.

A range of cell types, including keratinocytes, natural killer T-cells, plasmacytoid dendritic cells, and macrophages, produce cytokines that activate myeloid dendritic cells in the early stages of the pathogenesis of psoriasis. DNA-LL37 complexes, for example, induce plasmacytoid dendritic cells to release Interferon-alpha (IFN-α), which activates myeloid dendritic cells that in turn release Interleukin (IL)-12 and IL-23 upon stimulation. IL-12 induces naive T-cells to differentiate into TH1 cells.^2^ Intracellular IL-23 signaling is mediated by Tyk2-Jak2 and STAT3, which leads to the transcription of critical inflammatory mediators. These cytokines cause the proliferation of keratinocytes, the release of endothelial adhesion molecules, and an increase in the production of angiogenic mediators and immune cell infiltration in dermal lesions.^2^

The convoluted interplay between the immunological, natural, genetic, and epigenetic elements that underpin the etiology of this disease is still unclear. However, the advent of biological medicines that target the critical immune pathways involved in the etiopathogenesis of psoriasis, such as IL-17, IL-23, and Tumor Necrosis Factor (TNF), has changed the therapeutic landscape for severe forms of psoriasis. Regimens that include these novel agents can help patients with psoriasis reduce their disease burden and improve their quality of life. On the other hand, these targeted medicines are not completely curative and have constraints such as decreased efficacy over time, possible severe adverse effects, and poor clinical response in some people.^3^ Consequently, there is an increasing number of psoriasis patients identified who are refractory to systemic therapies. Therefore, there is an urgent need for state-of-the-art breakthroughs in the treatment of severe psoriasis and non-pharmaceutical strategies.

Stem cell therapy entails using somatic cells derived from either a donor (allogeneic) or an affected individual (autologous) to treat the underlying disease. Hematopoietic Stem Cells (HSCs) induced pluripotent stem cells, Mesenchymal Stem Cells (MSCs), and regulatory T-cells (Tregs) are examples of somatic cells already used or showing promise as stem cell therapy in psoriasis. Cardiovascular disorders, connective tissue diseases, and cancer are the three present applications of stem cell therapy worldwide, all of which have seen rapid clinical research and commercialization improvement. HSC Transplantation (HSCT) has attracted attention in terms of both ""transfer"" and ""cure"" of psoriasis after research that has focused primarily on musculoskeletal disease, systemic sclerosis, and multiple sclerosis. However, there has been little hypothesis-testing research on HSCT in psoriasis. Therefore, there appear to be both grounds and an incentive to investigate the use of stem cell therapy in psoriasis, particularly in individuals who have failed to respond to conventional treatments. This review discusses the epidemiology, pathogenesis, clinical presentation, diagnosis, and stem cell treatments for psoriasis.

## Methodology

**Figure 1:**
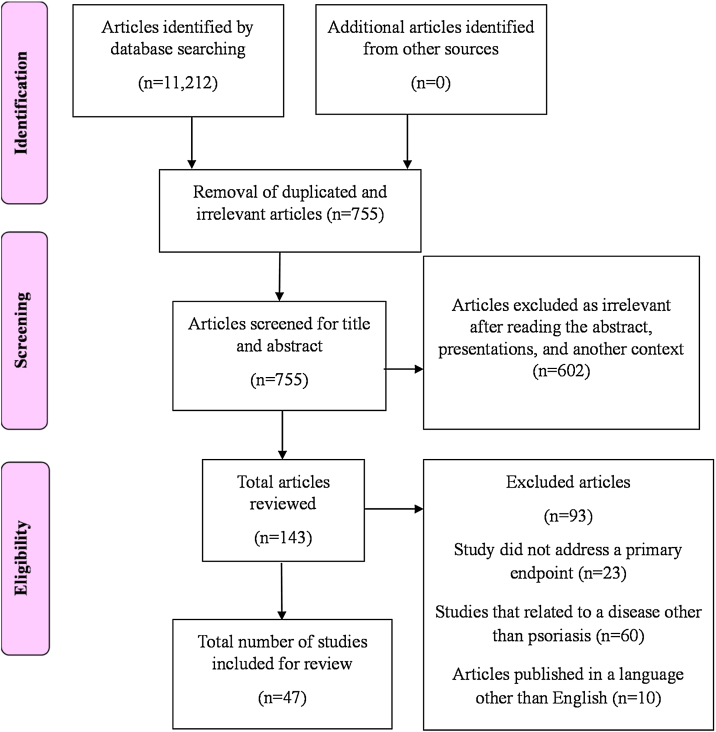
PRISMA flow chart.

The PubMed, Google Scholar, MEDLINE, Scopus, and Cochrane databases were electronically searched using appropriate key terms to identify articles on stem cell therapy as a potential treatment for psoriasis. The following terms were combined to search in MEDLINE and PubMed to identify the literature. Psoriasis AND “stem cell therapy” OR “regulatory T-cells” OR “hematopoietic stem cell transplantation” OR “mesenchymal stromal cells” OR “diagnosis”) AND (“cell therapy” OR (“psoriasis” AND “treatments”) OR “stem cells” OR (“psoriasis” AND “cell-based”). The initial literature search yielded 11,212 articles (Fig. 1). Articles published between January 1990 and July 2021 were reports that contained a description of stem cell therapy for psoriasis and were published in English. No restrictions were imposed according to the type of study design.

## Stem cell therapy in psoriasis

### Regulatory T-cells

Tregs regulate or suppress other immunocytes by modulating their responses to the endogenous environment and antigens, thereby helping to avoid autoimmunity and chronic inflammation. These functions are accomplished via inhibitory cytokines, such as IL-10, metabolism interference, modification of the maturation or functioning of dendritic cells, binding of lymphocyte-activation gene 3 to MHC class II molecules, and cytolysis (through granzyme A/B and perforin).^4^

Many treatments for psoriasis appear to boost the numbers of Tregs and their performance in patients with psoriasis. Anti-TNF therapy (particularly etanercept) increases Tregs and decreases the expression of IL-6 and IL-22.^5^ Increased numbers of Tregs may be associated with improved clinical outcomes, and infliximab stimulates a more divergent TCR repertoire.^6^ In one study, TNF blockade was found to increase the release of pro-inflammatory cytokines and Th17 activity and decrease numbers of Tregs and expression of Foxp3, resulting in worsening of the disease.^7^

Several biologics, including anti-IL-23 (p40-specific ustekinumab and p19-specific guselkumab) and anti-IL-17A (secukinumab), has been approved to treat psoriasis. There is other anti-IL 23 (risankizumab, guselkumab, briakinumab, tildrakizumab) and anti-IL17 (ixekizumab and brodalumab) approved and in the market.^8^ Anti-IL-17A and anti-IL-23 have been shown to enhance Foxp3+ cells. Therefore, anti-IL-17 treatment in psoriasis may restore a healthy Th17/Treg balance and thus reduce inflammation and ameliorate disease. In a recent Phase I–IIa trial, low-dose IL-2 was administered to patients with various autoimmune diseases. Patients with psoriasis showed increased Treg frequencies and improved body surface area and PASI scores.^9^ Therefore, a combination of low-dose IL-2 medication with a regimen that restores the functions of Tregs, e.g., phototherapy or sotrastaurin, is a promising therapeutic approach in psoriasis.

The stability and functionality of the Treg lineage depend on epigenetic programming. Histone Deacetylase (HDAC-1) is expressed in the tissues of people with psoriasis. Trichostatin-A, which inhibits the HDAC protein, has been found to shift Tregs towards an IL-17 phenotype.^10^ This finding suggests that HDAC inhibitors may also be beneficial in the treatment of psoriasis. By resetting the Th17/Treg ratio and decreasing the number of subsets of inflammatory T-cells, Treg-based cell therapies may have the ability to interfere with the pathogenesis of psoriasis despite their high cost and hazards.^11^

### Mesenchymal stem cells

**Figure 2:**
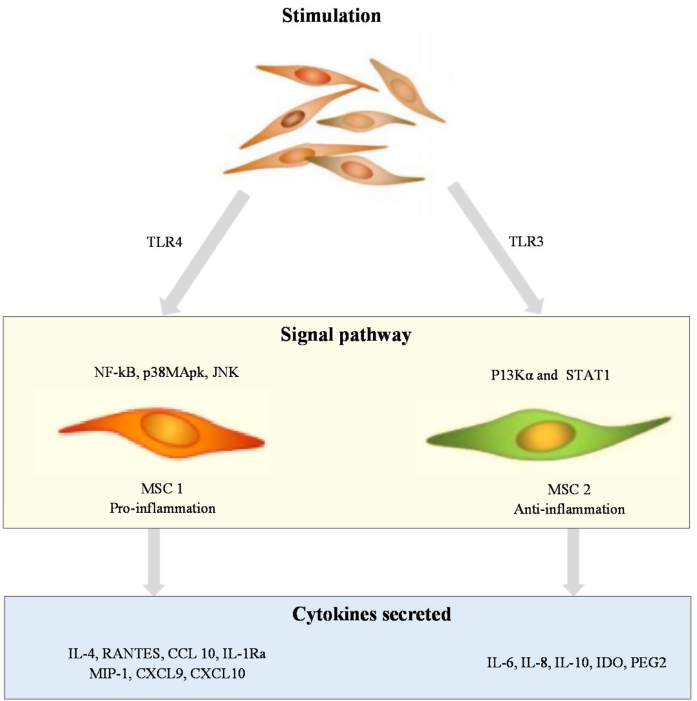
Role of mesenchymal stem cells in the pathogenesis of psoriasis.

MSCs are a type of pluripotent cell found in tissues of mesenchymal origin and are responsible for tissue regeneration. The role of MSCs in the pathogenesis of psoriasis is shown in Fig. 2. MSCs are distinguished by specific surface indicators and their ability to adhere to plastic substrates and develop into adipocytes, osteocytes, and chondrocytes in vitro. MSCs consist of various types of cells, and the most well-known are bone marrow-derived stromal stem cells. Other MSCs include umbilical cord MSCs, adipose-derived stem cells, and amniotic fluid stem cells, which are found in the stromal vascular fraction of adipose tissue. MSCs are being studied not only for their ability to regenerate cells but also for their immunomodulatory properties, given that they appear to modulate the release of lymphocyte-derived cytokines directly.^12^ According to a recent study, MSCs from the skin of patients with psoriasis show low differentiation capacity, low immunoregulatory function, and high HLA-I expression levels.^13^ In psoriatic skin, inhibition of lymphocytes appears to be impaired, likely because of the abnormal dermal MSCs, which result in abnormal cytokine secretion through circular RNA.^14^ Furthermore, MSCs from psoriatic plaques have been shown to stimulate the growth of keratinocytes, leading to epidermal thickening.^15^

Several studies have reported that MSCs play a significant role in determining the pleiotropic effects of biological therapy on keratinocytes and T-cells. According to Campanati et al., TNF-α inhibitors affect cutaneous MSCs, the cells most likely to be implicated in psoriasis.^16^ Several recent studies have investigated the use of MSCs as a possible therapeutic option for psoriasis (Table 1).^17^, ^18^, ^19^, ^20^, ^21^, ^22^, ^23^, ^24^, ^25^

**Table 1:** Studies of mesenchymal stem cells as a curative treatment for psoriasis.

Study number	Author(s)	Type of MSCs	Study type	Publication type
1	Owczarczyk-Saczonek et al.^17^	Bone marrow and umbilical cord MSCs	Preclinical, clinical	Review
2	Lee et al.^18^	Umbilical cord MSCs	Preclinical	Original research
3	Kim et al.^19^	Tonsil-derived MSCs	Preclinical	Original research
5	Campanati et al.^20^	Dermal MSCs	Preclinical	Original research
7	Kim et al.^21^	Embryonic stem cell derived MSCs	Preclinical	Original research
8	Imai et al.^22^	Amniotic fluid stem cells	Preclinical	Letter
9	Chen et al.^23^	Umbilical cord MSCs	Preclinical	Original research
10	Wang et al.^24^	Allogeneic gingival MSCs	Clinical	Case report
11	Chang et al.^25^	Human dermal derived MSCs	Clinical	Original article

MSCs, Mesenchymal Stem Cells.

MSCs play a role in innate and adaptive immunity in terms of immunomodulation. Their immune-enhancing activities are performed via synergy with immune cells, including neutrophils, dendritic cells, monocytes, natural killer cells, macrophages, B-cells, and T-cells via cell-to-cell encounters and paracrine activity.^26^ Several growth factors, chemokines, and cytokines are found in the MSC secretome, enveloped in extracellular vesicles; these include nitric oxide, indoleamine-pyrrole 2.3-dioxygenase, fibroblasts, and hepatocyte growth factors, IFN-ɣ, prostaglandin E2, TNF-α, and transforming growth factor-β1.^27^

Most of the favorable evidence concerning the use of MSCs in psoriasis comes from Phase I or II studies. To date, there has been no significant toxicity. However, more extensive controlled trials are needed to determine this treatment strategy's efficacy and long-term safety. Despite the growing body of knowledge and experience with MSCs in the clinical setting, cell dose and constancy differ between the trials reported to date, and the best regimen has yet to be identified.

### Hematopoietic stem cells

The favorable effects of HSCT in patients with lymphoma and leukemia and other autoimmune illnesses, including systemic lupus erythematosus, diabetes, rheumatoid arthritis, and multiple sclerosis, prompted investigations of HSCT in patients with psoriasis.^28^, ^29^, ^30^ In the last 25-years, extended remission of psoriasis has been documented in more than 30 patients who have undergone Bone Marrow Transplantation (BMT).^31^, ^32^ However, a small number of patients have developed psoriasis after receiving an allogeneic BMT or a blood transfusion from a donor with psoriasis.^31^ Details of the studies that have demonstrated remission of psoriasis after autologous HSCT are shown in Table 2.^33^, ^34^, ^35^, ^36^, ^37^, ^38^, ^39^, ^40^, ^41^

**Table 2:** Studies of remission of psoriasis after HSC transplantation.

Study number	Author(s)	Indication	Type of HSCT	Remission of psoriasis
1	Kishimoto^33^	Acute myeloid leukemia	Allogeneic	Two years of follow-up
2	Kaffenberger et al.^32^	Chronic myeloid leukemia	Allogeneic NST	Two years of follow-up
3	Kanamori et al.^34^	Chronic myeloid leukemia	Allogeneic BMT	2.5-years of follow-up
4	Mohren et al.^35^	Psoriatic arthritis	PBSCT	16-months
5	Woods and Mant^29^	Aplastic anemia	Allogeneic HSCT	12-months
6	Rossi et al.^36^	Acute aplastic anemia	Allogeneic BMT	Ten years of follow-up
7	Braiteh et al.^37^	Multiple myeloma	Autologous HSCT	>2 years of follow-up
8	Held et al.^38^	'Ewing's sarcoma	Autologous stem cell transplantation	15-months of follow-up
9	Mori et al.^39^	Myelodysplastic syndrome	Allogeneic BMT	Eight months of follow-up
10	Chen et al.^40^	Immunoglobulin light chain amyloidosis and psoriasis	Autologous HSCT	Follow-up for more than 7-years
11	Ugur and Gediz^41^	Psoriasis and multiple myeloma	Autologous stem cell transplantation	18-months of follow-up

BMT, Bone Marrow Tansplant; HSCT, Hematopoietic Stem Cell Transplantation; NST, Nonmyeloablative allogeneic Stem cell Transplantation; PBSCT, Peripheral Blood Stem Cell Transplantation.

According to the pathogenesis of psoriasis, T-cells can provoke the development of changes after a blood transfusion, but peripheral T-cells have a relatively short lifetime. Amelioration of psoriasis has been documented in patients who have undergone allogeneic (rather than autologous) HSCT, suggesting that hematopoietic stem cells could be the primary cause of the disease.^31^, ^42^ Mori et al. described the case of a 54-year-old male patient with a 10-year history of psoriasis who was treated with an allogeneic bone marrow transplant with preceding myeloablation with busulfan and cyclophosphamide because of a myelodysplastic syndrome, who achieved complete remission of psoriasis, which remained throughout the 8-month follow-up.^39^ The authors of that report stressed the need to eliminate autoreactive cells during allogeneic BMT and treatment with immunosuppressive medication to maintain remission. Similarly, Adkins et al. reported on a 55-year-old woman with refractory psoriasis and chronic myeloid leukemia who received an allogeneic BMT and achieved complete remission of her psoriasis,^28^ although the drugs used to prepare the patient for transplant (cyclophosphamide and busulfan) could have contributed to the rapid remission of psoriasis in this patient. Zurita et al. presented the findings of a trial that compared the effects of autologous HSCT (n = 30) with UVA photochemotherapy (n = 19) in patients with severe psoriasis. After obtaining bone marrow from the iliac crest and isolating the CD34+ fraction, autologous cells were administered intravenously as a single dose.^30^ The therapeutic effects were controlled for up to six months and compared with the effects of Photochemotherapy UVA (PUVA). PASI75 reached a statistically significant level in the group treated with stem cells, but no significant difference was observed compared to the effects of PUVA.^30^

### Advantages of stem cell therapy for psoriasis

The discovery of stem cell therapy as a feasible alternative treatment for refractory psoriasis has been serendipitous. Eedy et al. described a 35-year-old man who received allogeneic HSCs from his unaffected brother to treat acute myelomonocytic leukemia and was cured of severe, intractable psoriasis. The recipient was still clear of psoriasis five years after the transplant.^43^ The mechanisms underlying the effectiveness of allogeneic HSCT in psoriasis are still unknown. The immunosuppressive medications and immune ablation required for surgery are thought to decrease the number of autoreactive T-cells. The immune system is rebuilt with potentially non-reactive T-cell populations from donors who do not have psoriasis. A durable remission of psoriasis (for up to 20-years) has been reported in people who have undergone allogeneic HSCT rather than autologous HSCT.^32^

In a contrasting report, ""transfer"" of psoriasis was documented in a 40-year-old man with acute myeloid leukemia who received a syngeneic HSCT from his phenotypically identical twin brother with a 20-year history of severe psoriasis and psoriatic arthritis.^44^ The recipient sibling developed intractable psoriasis within ten days of the graft. Even after a second donation from the same donor, his condition continued with the onset of psoriatic arthritis. This case suggests that adoptive transfer can be used to transmit the cellular features of psoriasis. On the other hand, autologous HSCT does not fully alleviate psoriasis; relapses are common and can occur up to 10-years after transplantation. In one study, five of 11 patients with psoriasis who received autologous HSCT relapsed within two years, and one relapsed 13-years later.^45^ However, even when psoriasis recurs, it follows a more benign course than before the transplant. The myeloablative and lymphoablative effects of preconditioning and delayed immune reconstitution after transplantation are all factors to consider and are thought to be responsible for the remission of psoriasis after autologous HSCT. ^37^

Chen et al. explored the mechanism of action of umbilical cord-MSCs for psoriasis using the imiquimod mouse model and infusion of human umbilical cord-MSCs; this MSC significantly reduced psoriasis severity.^46^ Less generation of type I IFN by plasmacytoid dendritic cells was a significant characteristic of the response. Finally, Wang et al. used five infusions of allogeneic gingival MSCs, which have immunomodulatory and anti-inflammatory effects, to treat a 19-year-old man with severe plaque psoriasis that was recalcitrant to systemic regimens. The **patient's** psoriasis cleared after the sixth infusion, and the patient remained psoriasis-free three years later.^24^

## Conclusion

Psoriasis is an inflammatory skin disease associated with several comorbidities that have a significant impact on quality of life. The application of stem cells raises hopes for developing a new, safe, and effective therapy for psoriatic patients. Cell therapy in the form of MSCs may offer an attractive and safer option in psoriasis. Despite more data still being needed, MSCs could be a promising therapy for psoriasis.

## Financial support

None declared.

## Author's contributions

Piyu Parth Naik: Conceptualization, methodology, study conception & planning, critical literature review, preparation and writing of the manuscript, writing-original draft, writing-review & editing, approval of the final version of the manuscript.

## Conflicts of interest

None declared.
